# Time for a “Third Wave” of Malaria Activism to Tackle the Drug Stock-out Crisis

**DOI:** 10.1371/journal.pmed.1000188

**Published:** 2009-11-24

**Authors:** The PLoS Medicine Editors

## Abstract

The *PLoS Medicine* Editors discuss shortages of artemisinin-based combination therapy in Africa at the point of care and how these can be addressed.

“Probably no other disease in human history has been associated with social and political activism to the extent that the HIV epidemic has,” says the *AIDS Activism* Web site [1]. Such activism played a huge role in reducing the costs of antiretroviral drugs in developing countries [2]. Five years ago, one of us (GY) argued that similar activism was needed to raise awareness of shortfalls in global efforts to control malaria [3]. We believe there are now signs of an evolving “malaria activism,” which has resulted in two major successes.

The first wave of malaria activism highlighted the disparity between the massive burden of disease and the tiny amount of international development assistance dedicated to its control [4],[5]. This assistance amounted to just US$100 million in 2000, yet the World Health Organization (WHO) Commission on Macroeconomics and Health estimated that global malaria control would cost US$1.5–US$2.5 billion annually worldwide [5]. Advocacy by researchers, nongovernmental organizations, journalists, and editors helped motivate donors to increase their malaria commitments [5]–[7].

By 2007, according to a study by Bob Snow (KEMRI-Wellcome Trust, Kenya) and colleagues, international financing of malaria control had increased to around US$1 billion (40% came from the Global Fund to Fight AIDS, Tuberculosis, and Malaria) [7]. Although this upward trend in funding was a victory for “malaria activists,” there is still no room for complacency, since the study found insufficient funding to fully scale up malaria control worldwide. Commenting on the study, Anthony Kiszewski (Bentley University, USA) argued that while underfunding continues, the fashionable talk of eliminating malaria seems “quixotic at best” [8].

The second wave of malaria activism focused on exposing an inconvenient truth. A substantial proportion of the additional malaria funds was being spent on monotherapies (chloroquine, sulfadoxine-pyrimethamine), often ineffective in Africa because of parasite resistance [9],[10], rather than more efficacious artemisinin-based combination therapy (ACT). Activism by academics, journalists, and newspaper editors scored a further success: in 2004 the Global Fund reprogrammed its financing to spend more on ACT, while the United Nations (UN) health agencies pledged to discontinue support for the use of ineffective monotherapies [11],[12]. Since then, ACT has been scaled up across Africa [13].

These are big victories. But one benchmark of successful ACT scale-up is whether the drugs are available at the point of care [14]. One of us (GY) has just returned from a health reporting fellowship in East Africa [15], where he found that ACT “stock-outs” (shortages) were common. This observation is backed by empirical evidence from many African countries [14],[16],[17]. For example, in 2008, two years after Kenya introduced artemether-lumefantrine (AL) as first-line treatment for uncomplicated malaria, Beth Kangwana (KEMRI-Wellcome Trust, Kenya) and colleagues surveyed government health facilities to assess AL availability [14]. The drug comes in four different weight-specific blister packs. A quarter of the surveyed facilities had none of the four packs.

So it's time for a “third wave” of malaria activism to raise awareness of this ACT stock-out crisis. Interviewed at the KEMRI-Wellcome Trust Research Centre in Nairobi, Bob Snow, coauthor of the Kenyan survey [14], said the current situation is two steps forward, three steps back. “We abandoned chloroquine when it failed to cure one in four patients and was available everywhere,” he said. “We now have a drug that cures 100% of patients but is not available in one in four clinics.” Snow said that the stock-out crisis results from “inadequate ordering, distribution, and supply—it's a health systems issue.”

Chloroquine cost pennies and was being given presumptively to any child with fever. Supplying ACT to the right patient at the right time is more complex. The drug is expensive (around US$6–10 per treatment). Because it comes in four weight-specific packs, countries are essentially managing the supply of four drugs, not one. And there is growing pressure for public sector health facilities to abandon presumptive treatment and use a rapid diagnostic test (RDT) before prescribing ACT [18]. So the central medical supplies agency of each country is in effect managing the flow of five products.

A chain of events must occur for these products to be available at the point of care in public sector health facilities, starting with adequate funding. There are still funding shortfalls to cover a country's essential stocks, including buffer stocks. Interviewed in Nairobi, Elizabeth Juma, who heads the Division of Malaria Control in Kenya's Ministry of Health, said: “For a buffer, you need nine months of stock at a cost of about $6m.” It's critical that Kenya gets buffer stocks, she said. “We need to quantify and request funds for that.”

If a developing country has adequate funding, which in most cases comes via the Global Fund [19], the next step is to procure sufficient high-quality drug. Initially, there was only one fixed-dose ACT (AL) and countries usually obtained it via the WHO at cost price for public sector use (as set by a formal agreement with the manufacturer). As new suppliers and new types of ACT became available (WHO prequalified ACTs are listed at [20]), countries used a wider range of procurement procedures. Of these, national procurement by open tendering has been increasingly encouraged by international funding agencies because it fosters country ownership of the process, and the competition between ACT suppliers can bring down drug prices. But the process can face administrative and bureaucratic delays and hurdles, particularly in countries with under-resourced health and regulatory systems [14]. Drug companies have sometimes attempted to sway the tendering process in their favor, such as by spreading misinformation about their competitors, which has resulted in significant delays in procurement (Andrea Bosman, personal communication).

After procurement, the drug must be distributed from central medical stores to peripheral health facilities. In countries with weak health information systems, stock-outs can occur because stock flows aren't being adequately tracked. There may be no data on how many patients are being treated or which facilities are running low on ACT. Difficulty in predicting ACT requirements centrally and peripherally can lead to an underestimation of need. “The whole science of commodities prediction is underdeveloped,” said Snow.

To add further complexity, the cost of ACT purchased in the private sector is set to fall to US$0.2–0.5 per treatment, thanks to a global subsidy, the Affordable Medicines Facility–malaria (AMFm) [21]. In a pilot study in Tanzania, introducing subsidized ACT was associated with a rapid increase in the proportion of people accessing the drug from private shops [22]. While this study suggests that the AMFm can improve private sector access to ACT, Suerie Moon and colleagues warn that the subsidy could undermine public-sector ACT provision (for example, in the event of ACT shortages, the AMFm would be competing for limited ACT stock) [23]. The AMFm also introduces a confusing double standard into malaria management. On the one hand, there's a push for the public sector to use RDTs and educate people that fevers *aren't* always due to malaria. On the other, the AMFm offers ACT over the counter, sold by private providers who have an incentive to maximize sales and no training or facilities to use RDTs. The implicit message in the private sector: your child with fever *has* malaria and needs presumptive over-the-counter ACT.

What might the “third wave” of malaria activism look like? Publicizing ACT stock-outs is a crucial element. Two examples are Sarah Boseley's news feature on stock-outs in Uganda [24] and the launch of Africa's Stop Stock-outs campaign [25]. Advocacy must also focus on ensuring that the ACT supply chain is made less vulnerable. Donors must step up to fill the funding gaps identified by Snow and colleagues [7]. Endemic countries need technical assistance from the WHO and the Global Fund to build national capacity for ACT procurement, and to develop stock management information systems. Pilot projects are needed to test the efficacy of these information systems (Roll Back Malaria, for example, is piloting the “SMS for Life” project, which uses text messages to track ACT stocks [26]). The impact of the AMFm on public sector provision of ACT needs to be monitored. And there needs to be a major investment into research on forecasting ACT requirements internationally, nationally, and in peripheral clinics and on managing commodities. Such research doesn't sound as exciting as, say, developing a new malaria vaccine or medicine, but it is crucially important in preventing stock-outs and thus malaria deaths—malaria activism's ultimate goal.

## Abbreviations

ACT: artemisinin-based combination therapy
AL: artemether-lumefantrine
AMFm: Affordable Medicines Facility-malaria
RDT: rapid diagnostic test

## References

[1] AIDS Activism Web site (2009). University at Albany HIV/AIDS Information Center.. http://www.albany.edu/sph/AIDS/activists.html.

[2] Smith RA, Siplon PD (2006). Drugs into bodies: Global AIDS treatment activism.

[3] Yamey G (2004). Roll Back Malaria: A failing global health campaign.. BMJ.

[4] Rowe AK, Rowe SY, Snow RW, Korenromp EL, Schellenberg JR (2006). The burden of malaria mortality among African children in the year 2000.. Int J Epidemiol.

[5] Narasimhan V, Attaran A (2003). Roll Back Malaria? The scarcity of international aid for malaria control.. Malaria J.

[6] Malaney P, Spielman A, Sachs J (2004). The malaria gap.. Am J Trop Med Hyg.

[7] Snow RW, Guerra CA, Mutheu JJ, Hay SI (2008). International funding for malaria control in relation to populations at risk of stable *Plasmodium falciparum* transmission.. PLoS Med.

[8] Kiszewski AE (2008). Divergent goals and commitments in global malaria intervention.. PLoS Med.

[9] Yamey G (2003). Malaria researchers say global fund is buying “useless” drug.. BMJ.

[10] Attaran A, Barnes KI, Curtis C, d'Alessandro U, Fanello CI (2004). WHO, the Global Fund, and medical malpractice in malaria treatment.. Lancet.

[11] Yamey G (2004). Health agencies end in-fighting on malaria.. BMJ.

[12] Butler D (2004). Global Fund changes tack on malaria therapy.. Nature.

[13] Bosman A, Mendis KN (2007). A major transition in malaria treatment: The adoption and deployment of artemisinin-based combination therapies.. Am J Trop Med Hyg.

[14] Kangwana BB, Njogu J, Wasunna B, Kedenge SV, Memusi DN (2009). Malaria drug shortages in Kenya: A major failure to provide access to effective treatment.. Am J Trop Med Hyg.

[15] Yamey G (2009). Reporting from East Africa and Sudan [blog post].. http://speakingofmedicine.plos.org/2009/07/02/reporting-from-east-africa-and-sudan.

[16] Zurovac D, Ndhlovu M, Sipilanyambe N, Chanda P, Hamer DH (2007). Paediatric malaria case-management with artemether-lumefantrine in Zambia: a repeat cross-sectional study.. Malar J.

[17] Zurovac D, Tibenderana JK, Nankabirwa J, Ssekitooleko J, Njogu JN (2008). Malaria case-management under artemether-lumefantrine treatment policy in Uganda.. Malar J.

[18] D'Acremont V, Lengeler C, Mshinda H, Mtasiwa D, Tanner M (2009). Time to move from presumptive malaria treatment to laboratory-confirmed diagnosis and treatment in African children with fever.. PLoS Med.

[19] Cohen JM, Singh I (2008). Global Forecast of ACT Demand. Boston: Clinton Foundation HIV/AIDS Initiative.. http://www.clintonfoundation.org/what-we-do/clinton-hiv-aids-initiative/information-center-.

[20] WHO (2007). Antimalarial medicines available for procurement by WHO.. http://apps.who.int/malaria/pages/performance/antimalarialmedicines.html.

[21] Affordable Medicines Facility – Malaria (2009). FAQ page.. http://www.theglobalfund.org/documents/amfm/AMFmFAQs_en.pdf.

[22] Sabot OJ, Mwita A, Cohen JM, Ipuge Y, Gordon M (2009). Piloting the global subsidy: The impact of subsidized artemisinin-based combination therapies distributed through private drug shops in rural Tanzania.. PLoS ONE.

[23] Moon S, Pérez Casas C, Kindermans J-M, de Smet M, von Schoen-Angerer T (2009). Focusing on quality patient care in the new global subsidy for malaria medicines.. PLoS Med.

[24] Boseley S (2009). In Katine, a Coke is easy to buy. Medicine isn't.. http://www.guardian.co.uk/katine/2009/aug/20/katine-malaria-medicine-aid.

[25] (2009). Stop Stockouts.. http://stopstockouts.org/.

[26] Roll Back Malaria (2009). SMS for life.. http://www.rollbackmalaria.org/psm/smsWhatIsIt.html.

